# The advantages and disadvantages of horizontal gene transfer and the emergence of the first species

**DOI:** 10.1186/1745-6150-6-1

**Published:** 2011-01-03

**Authors:** Aaron A Vogan, Paul G Higgs

**Affiliations:** 1 Origins Institute, McMaster University, Hamilton, Ontario L8 S 4M1, Canada

## Abstract

**Background:**

Horizontal Gene Transfer (HGT) is beneficial to a cell if the acquired gene confers a useful function, but is detrimental if the gene has no function, if it is incompatible with existing genes, or if it is a selfishly replicating mobile element. If the balance of these effects is beneficial on average, we would expect cells to evolve high rates of acceptance of horizontally transferred genes, whereas if it is detrimental, cells should reduce the rate of HGT as far as possible. It has been proposed that the rate of HGT was very high in the early stages of prokaryotic evolution, and hence there were no separate lineages of organisms. Only when the HGT rate began to fall, would lineages begin to emerge with their own distinct sets of genes. Evolution would then become more tree-like. This phenomenon has been called the Darwinian Threshold.

**Results:**

We study a model for genome evolution that incorporates both beneficial and detrimental effects of HGT. We show that if rate of gene loss during genome replication is high, as was probably the case in the earliest genomes before the time of the last universal common ancestor, then a high rate of HGT is favourable. HGT leads to the rapid spread of new genes and allows the build-up of larger, fitter genomes than could be achieved by purely vertical inheritance. In contrast, if the gene loss rate is lower, as in modern prokaryotes, then HGT is, on average, unfavourable.

**Conclusions:**

Modern cells should therefore evolve to reduce HGT if they can, although the prevalence of independently replicating mobile elements and viruses may mean that cells cannot avoid HGT in practice. In the model, natural selection leads to gradual improvement of the replication accuracy and gradual decrease in the optimal rate of HGT. By clustering genomes based on gene content, we show that there are no separate lineages of organisms when the rate of HGT is high; however, as the rate of HGT decreases, a tree-like structure emerges with well-defined lineages. The model therefore passes through a Darwinian Threshold.

**Reviewers:**

This article was reviewed by Eugene V. Koonin, Anthony Poole and J. Peter Gogarten.

## Background

Traditionally, genetics is the study of vertical transmission of genes from parents to offspring. Since complete genomes have become available, it has become clear that, in addition to vertical inheritance, there is a substantial rate of horizontal gene transfer (HGT) from unrelated individuals, at least in prokaryotes. According to the principle of natural selection, if a gene increases the fitness of an individual, that individual will have a larger number of offspring on average, and the offspring will inherit the beneficial gene. Hence, the frequency of the beneficial gene will increase in the population. It is therefore clear that vertical inheritance of genes allows natural selection to occur. In contrast, if a gene is acquired horizontally, there is no guarantee that it increased the fitness of the previous individual. Although there is the potential to gain useful new genes by HGT, there is also the possibility of acquiring useless or harmful genes. HGT is a risky evolutionary strategy, whereas vertical inheritance is safe because the genes have been tried and tested in the parent. The gain of a gene by HGT depends on many processes inside the receiving cell. Therefore, cells can potentially evolve to increase or decrease their rate of acceptance of horizontally acquired genes. In this paper, we ask if HGT is on average beneficial to the organism and whether selection will act to increase or decrease its rate.

If HGT is frequent, the traditional picture of the tree of life is no longer valid [1,2]. Although it is possible to construct a tree from the concatenated sequences of a small number of highly conserved genes that are found in all organisms [3], this tree is not representative of the majority of the genes in most genomes [4]. Nevertheless, even if HGT events are reasonably common, the signal of the underlying tree is still discernible as a central trend [5,6]. Gene trees tend to be slightly discordant, but not completely randomized.

Woese has argued that HGT was particularly important in the early stages of life on Earth [7]. In his view, separate lineages of species did not exist, but rather the entire community of organisms co-developed and evolved as one interconnected network. When individuals attained a certain degree of complexity, the likelihood of a gene gained by HGT being beneficial would decrease, and the net HGT rate would decrease. At this point, the population would cross what Woese coined, the "Darwinian Threshold". After this point, separate, tree-like evolutionary lineages could emerge. This picture seems to be compatible with recent analysis of the degree of phylogenetic consistency between gene trees [6]. Recent branches of the tree tend to be fairly consistent across genes, whereas there is much higher inconsistency deep within the tree at the time of emergence of the major prokaryotic groups. In this paper, we consider a simple evolutionary model that explains why we would expect a Darwinian Threshold to occur.

The most obvious benefit to HGT is that a cell can acquire a beneficial gene that arose in another cell. The origin of new beneficial genes is presumably extremely rare, and stealing a gene from a neighbor should be much quicker than waiting to evolve it independently. Secondly, cells may lose genes by deletion or by accumulation of deleterious mutations. Early cells may have had genes as separate molecules or as very short chromosomes, rather than as large fully-linked chromosomes as in modern prokaryotes. Inaccurate segregation would be another problem that would lead to gene loss. If replication accuracy was poor, accidental gene loss would limit the size and complexity of genomes that could evolve. In this case, HGT would be beneficial, because it would enable a cell to regain a gene that it had lost from another member of the population. The set of genes that could be maintained by the whole population, known as the pan-genome [8,9] would be larger than the set contained in any one individual.

HGT also has many potential disadvantages. The simplest of these is that increasing the genome size will increase the replication time of the organism, so there will be selection against genomes that contain large, non-functional sections. A sequence acquired by HGT may be non-functional because it is non-coding, because it is a duplicate copy of a gene already in the cell, or because it only functions in the presence of other genes that are not contained in the recipient cell. If a newly-inserted gene is expressed, it imposes a cost of transcription and/or translation that will be detrimental to the cell if the gene product has no useful function. Furthermore, if a horizontally acquired sequence is inserted randomly into a genome, it may disrupt the existing gene at the point of insertion. The new gene product might also interfere with the function of existing molecules in the cell. Finally, worse than any of the above, the new gene may be a selfish replicator, *e.g*. a transposable element that duplicates itself many times within the host genome or a virus that destroys the infected cell. So, evolutionarily, it should pay a cell to 'think twice' before accepting a foreign gene into its genome.

A considerable amount is known about the way HGT occurs in modern prokaryotes. Transformation is the process by which cells import fragments of DNA from the surrounding medium [10,11]. Once foreign DNA is inside the cell, there is some possibility that it may recombine with the cell's DNA and become part of the genome. Transformation requires expression of membrane proteins involved in DNA transport, and is thus an active process. Not all types of bacteria are competent for DNA import, and competent species do not necessarily turn on their DNA import machinery at all times. Thus, it is clear that cells can evolve to control their rate of import of foreign DNA and this will influence the likelihood that horizontally transferred genes end up in the genome.

The other two mechanisms of HGT in modern cells are conjugation, in which a mobile element such as a plasmid actively transfers a copy of itself into another cell, and transduction, in which a gene from a host cell is packaged inside a virus and transferred to another host along with the virus genes [11,12]. These transfer mechanisms are controlled by genes on the mobile elements themselves; hence the rate of acquisition of horizontally transferred genes by these mechanisms is less easily controlled by the recipient cell than it is for transformation. Nevertheless, recipient cells can still influence on the fate of the foreign DNA once it has entered the cell by controlling the rate of recombination [13,14] or evolving restriction enzymes that cleave foreign DNA [15]. The net rate of HGT is adjustable by all these means and is therefore subject to evolution. There are also mechanisms by which cells can silence the expression of foreign genes that have become inserted into the genome [16]. This avoids some of the potentially harmful consequences of HGT, even though it does not prevent the insertion event.

In the context of all the above factors that influence the occurrence of HGT and its costs and benefits, we now present a simple theoretical model to determine under which circumstances HGT is advantageous and to address the consequences of the changing rate of HGT from the earliest organisms to modern prokaryotes.

## Methods

The evolutionary model is defined in the following way. The genome of each cell in the population is a list of integers, where each integer is a label for a type of gene. Some genes may be present in duplicate copies on the same genome, so that the number of types of genes on the genome, *n_types_*, may be less than the total number of genes on the genome, *n*. There is a selective advantage, *s*, for each different type of gene on the genome, and a cost, *c*, for every gene (including duplicates). The fitness of an individual genome is defined as *w *= (1+*sn_types_*)/(1+*cn*). We suppose that *s *>*c*, so that non-duplicated genes are beneficial, but duplicate copies are deleterious.

We consider the evolution of a population of *N *individuals. At each time step, one genome is chosen to replicate with a probability proportional to its fitness, and one individual is chosen to die with equal probability. A generation is N birth/death events. The genome of each new individual is copied from its parent. Each gene is lost due to gene deletion with probability *v *and is successfully copied to the offspring with probability 1-*v*. With probability *u*, a single new type of gene arises in the offspring. Each gene type arises only once, and is given a unique integer label to distinguish it from previous types. The new genome may then acquire genes by HGT. Each gene gained is a copy of a random gene in a random member of the population. This is intended to model uptake of genes by transformation. The DNA fragments available for uptake will come from cells that have recently died; hence, they should be similar to genes in the current population. The number of genes gained by the new genome is a random integer, *k*, chosen from a Poisson distribution, *P*(*k*) = *h^k ^*exp(-*h*)/*k*!. The parameter *h *is the mean number of genes gained by HGT per genome. Note that *k *is usually 0 or 1, but can be more than 1 if *h *is large.

We use a definition of distance between genomes that has successfully been used to construct phylogenetic trees from real bacterial genomes [17]. For any pair of individuals *i *and *j*, let *n_i _*and *n_j _*be the number of types of gene in the two genomes and let *n_ij _*be the number of types of genes shared by both genomes. The maximum number of genes that could be shared is equal to the smaller of the two genome sizes, min(*n*_*i*_, *n*_*j*_). Therefore, we define the similarity as *S_ij _*= *n_ij_*/min(*n_i_*, *n_j_*), and the inter-genomic distance as *d_ij _*= -ln(*S_ij_*).

Before presenting the results obtained with this model, it is worth considering a few points that are not included in the model. Firstly, we do not include variations at the level of single nucleotide mutations within a gene. Achieving a high accuracy of replication at the sequence level is clearly an important challenge for early organisms, and other types of models such as error-threshold models [18] address this point Relatively accurate sequence replication must have been achieved relatively early in evolution before genomes that contained many genes could evolve. The only parameter in the model that is related to genome replication accuracy is the gene loss rate, *v*. It would also be possible to introduce deleterious mutations which would create non-functional genes. A deleterious mutation would be more detrimental than a gene deletion in this model because the benefit of the gene, *s*, would be lost but the cost, *c*, would still be present, whereas with a deletion, both *s *and *c *would be lost. Qualitatively, we expect gene deletions and deleterious mutations to have a similar effect in this model; therefore, for simplicity, we include only the deletion rate. Note that deletions are essential in this model, because otherwise genome sizes would increase indefinitely.

There has been a lot of theoretical work on self-replicating molecular systems prior to the origin of organisms with genomes in the modern sense - in particular, the hypercycle theory [19] gives a mathematical model for autocatalytic reaction cycles. A model of the origin of autocatalytic cycles involving RNA molecules has also been proposed recently [20]. There have also been a number of studies of protocells at the level of 'bags of genes', in which each gene is assumed to be a separate molecule. This raises the issues of how a cell avoids loss of important genes by inaccurate segregation and how it avoids being over-run by selfish replicators. Theories based on the stochastic corrector model [21-25] go a long way to address these issues. However, in our view, these questions are relevant at an earlier stage of evolution than that which we consider here. We are most interested in the stage of the emergence of the major lineages of prokaryotes. It is likely that cells had already reached a level of complexity with hundreds or even thousands of genes by this stage - *i.e*. comparable to modern prokaryotes. It is difficult to conceive of cells reaching this level of complexity unless they had already evolved linked chromosomes and some kind of chromosomal segregation that is much better than random. Therefore our model is based on replication of a bacterial chromosome with the possibility of gene loss by deletion, rather than on random segregation of independent genes.

## Results

### Determination of the optimal rate of HGT

For all the examples shown here, we keep *N *= 500, *s *= 0.1 and *c *= 0.01. The rate of origin of new genes is presumed to be slow. In all these examples we fix *u *= 1/*N *= 0.002, so that there is only one new gene in the whole population per generation. Lowering this rate further would slow down the time scale of the simulations unnecessarily, but would not qualitatively change the predictions of the model. The key variables to be studied in the simulation are the gene deletion rate, *v*, and the horizontal transfer rate *h*. Simulations were begun with a population of identical individuals having one gene each. For each combination of parameters, a simulation was run for many generations until a stationary state was reached. Mean quantities were then determined over 500,000 generations in the stationary state.

**Figure 1:**
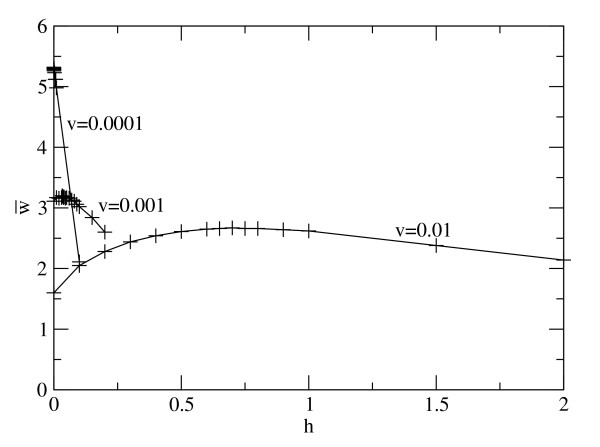
**Mean fitness of the population versus HGT rate, *h*, for three different rates of gene deletion, *v***.

Figure 1 shows the mean fitness $\bar{w}$ as a function of *h *for three different values of *v*. For the largest value, *v *= 0.01, there is an optimum close to *h = *0.6. When *v *is reduced to 0.001, the optimum reduces to *h *= 0.035. For the smallest deletion rate, *v *= 0.0001, the optimum horizontal transfer rate is *h *= 0 (or is not distinguishable from zero in our simulation). The case *v *= 0.01 is intended to model the situation in early cells with very inaccurate replication. Note that *v *is *per gene*. A genome of 100 genes will lose one gene per generation on average. A high rate of HGT is required to balance this loss, *i.e. h *is of order 1 *per individual*. The rate of gain of genes by HGT is much larger than the rate of gain of genes by *de novo *evolution, which is only *u *= 0.002 per individual in the simulation (and presumably even smaller in reality).

**Figure 2:**
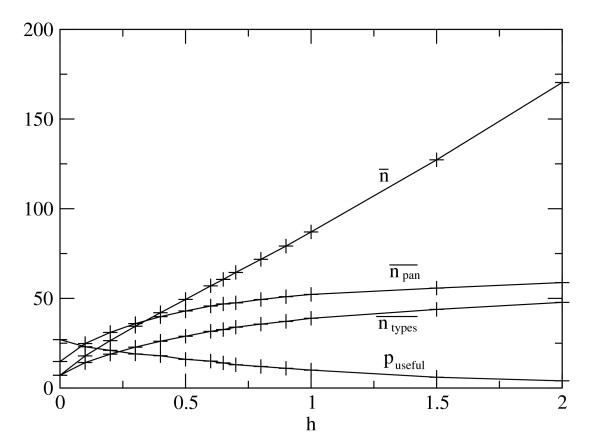
**Mean values of properties of the population as a function of *h *for the case of high gene deletion rate, *v *= 0.01**. $\bar{n}$, number of genes per individual; $\bar{n_{types}}$, number of different types of gene per individual; $\bar{n_{pan}}$, number of different types of gene in the whole population; *p_useful_*, probability that a horizontally transferred gene is useful to the receiving organism.

Figure 2 helps to explain why there is an optimal *h*. The mean number of genes per individual, $\bar{n}$, increases with *h *because gain of genes by HGT balances gene loss. The mean number of types of gene per individual, $\bar{n_{types}}$, also increases with *h*, but not as quickly as $\bar{n}$. The difference between these two curves is the number of duplicate genes per individual. This becomes very large when *h *is high. Figure 2 also shows the size of the pan-genome: $\bar{n_{pan}}$ is the mean number of types of gene in the whole population. Clearly $\bar{n_{pan}}$ is greater than $\bar{n_{types}}$. A gene acquired by HGT is only useful if it is different from the genes already in the genome. From the simulations, the probability *p_useful _*that the acquired gene is useful was found to decrease from about 25% when *h *= 0 to only a few percent for large *h*. Thus, if *h *is small, genomes remain of limited size. Larger, higher-fitness genomes can be maintained if *h *is larger. However, if *h *is too large, HGT causes the build-up of large numbers of duplicate genes that reduce the fitness. When genome replication is more accurate (smaller *v*), the optimum *h *is reduced, and is found to be zero for very small *v*. In the latter case, large, high-fitness genomes can be accurately replicated and are maintained in the population by selection, even in absence of HGT. If *h = *0, there are no duplicate genes. HGT does more harm than good in this situation because it creates duplicates.

### Evolution of the rate of HGT

From the above results, we would expect that cells would evolve towards high or low rates of HGT depending on whether the gene loss rate is high or low. In order to show this, we allowed *h *to be a variable property of individual cells. Simulations were performed in which each new cell inherited the *h *value of its parent, but with a small probability, the offspring *h *was mutated to be slightly higher or lower than the parent. When *v *= 0.01, the mean HGT rate of the population, evolved towards a stable intermediate rate around $\bar{h}=0.4$. This is less than the value of *h *= 0.6 at which the peak in fitness occurs in Figure 1. Similarly, when *v *= 0.001, the mean HGT rate evolved towards approximately $\bar{h}=0.01$, which is less than the value of 0.035 where the peak fitness occurs. For the smallest deletion rate, *v *= 0.0001, where the optimum in Figure 1 is at *h *= 0, it was found that $\bar{h}$ evolved towards a very low value that depended on the details of the way *h *was mutated between parents and offspring.

The fact that $\bar{h}$ does not evolve straightforwardly to the position of the peak fitness in the first two cases shows that evolution does not automatically optimize the mean fitness of the population. The most likely reason why the dynamics leads to smaller than optimal *h *is that if a low *h *value arises on a genome that has higher than average fitness, this low *h *value is beneficial in the short term because it maintains the integrity of this genome. Therefore the new *h *will spread, even though the mean fitness of the population will decrease in the long term if all individuals have the new *h *value. On the other hand, if a higher than optimal *h *value arises on a fitter than average individual, the descendants of this individual will acquire large numbers of duplicate genes and will not retain a high fitness. Therefore a too-large *h *value is unlikely to spread.

**Figure 3:**
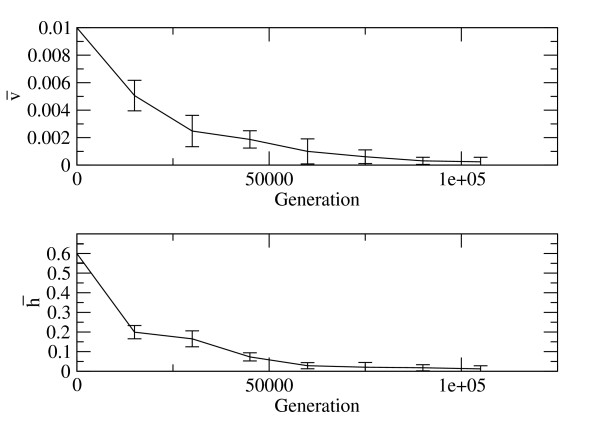
**Variation of mean deletion rate, $\bar{v}$, and mean HGT rate, $\bar{h}$, as a function of time in simulations in which both quantities are heritable**. Error bars show standard deviations over five runs.

What is most important for the current argument is that $\bar{h}$ does indeed evolve towards smaller values when *v *is smaller. It is particularly interesting to see what happens when both *v *and *h *are allowed to vary and be inherited from parent to offspring. Gene deletion is on average deleterious in this model; therefore we expect variants with lower values of *v *to be selected. We began with individuals with *v *= 0.01 and *h *= 0.6, representing early cells with inaccurate replication and frequent HGT. Figure 3 shows mean values $\bar{v}$ and $\bar{h}$ as a function of time. These are averaged over individuals in the population and over five independent runs of the simulation. Error bars show standard deviations across the simulation runs. The model shows that $\bar{v}$ evolves towards very low values because highly accurate replication is advantageous. It is also seen that $\bar{v}$ evolves towards very low values because lower *h *is favoured when *v *is lower. Limiting values of $\bar{v}$ and $\bar{h}$ depend on the way *v *and *h *are mutated between parents and offspring, and both would tend to zero if only selection were operating.

### Emergence of Evolutionary Lineages

**Figure 4:**
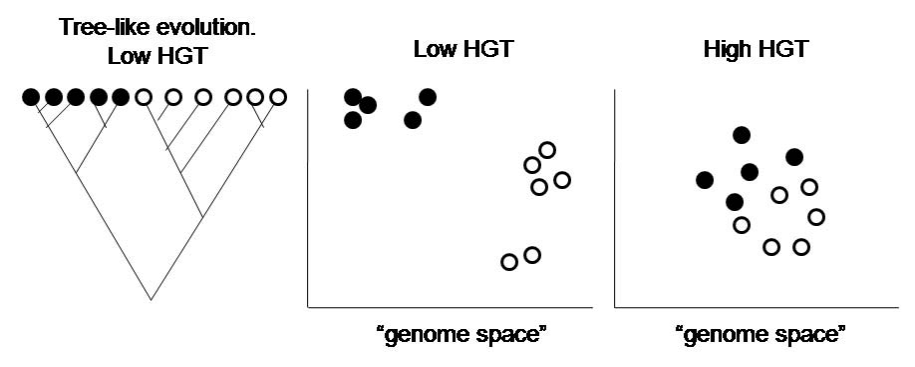
**Clustering individuals according to similarity of genomes**.

As discussed in the background section, evolution is expected to be tree-like in absence of HGT, but will become a tangled web if HGT is frequent. If there is an evolutionary tree, then it should be possible to cluster genomes according to their similarity. The basal split in the tree defines the two largest scale clusters of genomes, indicated schematically as black and white points in Figure 4. Genomes in the same cluster should be closer to one another in genome space than they are to genomes in the other cluster. If the tree is well-defined, there will also be sub-clusters nested hierarchically within the larger clusters (as in the centre of Figure 4). On the other hand, if there is a high HGT rate, then there will not be a clear way to split genomes into clusters. There will be an amorphous cloud of points in genome space, and although some genomes will be slightly closer to one another than others, any attempt that we make to split the population into clusters will be rather ill-defined and unsatisfactory (as on the right of Figure 4).

We will now use the simulations of Figure 3 to show that clusters of genomes are not well defined in our model when *h *is high, but that they become well defined as *h *decreases during the progress of the simulation. Our model therefore demonstrates the emergence of separate evolutionary lineages over time, *i.e*. it goes through Woese's Darwinian threshold [7].

For each set of genomes generated by the model, we calculated the distance matrix between all pairs of genomes, as described in Methods. This matrix was used as input to the standard UPGMA method of hierarchical clustering. Only the two largest-scale clusters were used, *i.e*. the two clusters that remain at the penultimate step before the root is reached. Using these two clusters, we measured *d_1_*, the mean distance between pairs of individuals in the same cluster, and *d_2_*, the mean distance between pairs of individuals in different clusters. The clustering ratio, *R = d_2_/d_1_*, was used as a measure of the extent of separation of these large-scale clusters. The higher the clustering ratio, the more clearly defined is the split at the base of the evolutionary tree.

Rows 1-8 of Table 1 correspond to values of $\bar{v}$ and $\bar{h}$ that arose at regularly spaced time intervals in Figure 3. Row 9 corresponds to the smallest *v *that was used in Figure 1 combined with *h *= 0. As *R *fluctuates a lot between populations for any given parameters, it is necessary to generate many populations for each set of parameters. Simulations were performed with *v *and *h *fixed at each of the combinations shown in the table. Genome data was printed out at well-spaced intervals, thus generating 100 independent populations for each parameter set. *R *was calculated for each population. Mean *R *values are shown in Table 1. For the highest HGT rate (Row 1), *R *is only 1.54. The clustering algorithm always produces a result even if the input data matrix is very far from tree-like. This means that *R *is bound to be greater than 1. However, this low value of *R *indicates that the basal split of the tree is poorly defined (as on the right of Figure 4). Table 1 shows that as *h *decreases, *R *becomes much larger. Thus, the basal split is very clearly defined in these latter cases (Rows 8 and 9): separate lineages have emerged.

**Table 1:** Dependence of the clustering ratio, R, on rates of deletion, v, and horizontal transfer, h.

Row	Generation	*v*	*h*	*R *(mean ± std. err.)
1	0	0.0100	0.600	1.54 ± 0.02

2	15000	0.0051	0.200	1.95 ± 0.05

3	30000	0.0025	0.165	2.35 ± 0.07

4	45000	0.0019	0.073	2.59 ± 0.09

5	60000	0.0010	0.028	3.06 ± 0.13

6	75000	0.0006	0.021	3.85 ± 0.33

7	90000	0.0003	0.018	4.26 ± 0.27

8	105000	0.0002	0.012	4.66 ± 0.38

9	----	0.0001	0.000	7.46 ± 0.87

## Discussion

Among those who study the evolutionary consequences of HGT, there is a tendency to presume that it is automatically advantageous. According to Gogarten *et al. *[1], "it is not clear that any evolved barriers to intergroup exchange (other than those effective against lethal viruses and parasitic genetic elements) should exist in prokaryotes". They further argue that "even the most promiscuous prokaryotes experience recombination much less frequently than they reproduce", and therefore "there is very little selective advantage in preventing such rare interspecific exchange". Our model shows that this is not always true, and that we would expect barriers to HGT to evolve in cases where there is accurate genome replication and large genomes can be efficiently transmitted vertically.

The simple fitness definition in this model is actually as favorable as possible to HGT. Every non-duplicate gene is assumed to be beneficial, independently of its context, whereas, more realistically, one would often expect the function of a gene to depend on the presence of specific other genes with which it interacts. The only disadvantage to HGT in the model is the cost of gene duplicates. There is no possibility of the inserted gene disrupting an existing gene, and there are no selfish replicators. If any of these additional features were added to the model, HGT would be less advantageous than in the case considered. Therefore, our conclusion that HGT is disadvantageous when there is accurate genome replication is robust. Woese [7] argued that vertical inheritance would become more important when cell design became more complex and a more integrated cellular organization emerged. It would be possible to change the fitness function in our model to include networks of interacting genes, in which case we would expect large, high-fitness genomes to have complex interactions between genes. However, it does not matter whether the larger genomes have high fitness because of independent beneficial genes (as in the current model) or because of complex networks of integrated genes. The essential point is that large genomes can only evolve if replication is accurate, and HGT becomes unfavorable when replication is sufficiently accurate.

Poole [26] has raised some interesting questions about whether selection acts at the level of genes or cells in the earliest organisms. Modern bacterial genomes contain sets of genes that interact with one another and function as a team. For genomes like this to evolve, teams of genes have to stay together for long enough for natural selection to increase the frequency of high-fitness teams. At the other extreme, one could envisage a world with such a high rate of gene exchange that every cell contained a random set of genes that was uncorrelated with sets of genes in previous cells. A way to simulate this situation in a model would be to create a new cell by selecting each gene independently from a different parental cell. In this extreme limit, selection would clearly act only at the gene level. We emphasize, however, that our model is far from this extreme. Even with the highest deletion rate considered in Figure 1 the optimum *h *is only of order 1, and there are of order 100 genes in the genome. Thus most genes come from a single parental cell, and genomes contain sets of genes that are correlated with the sets of genes on ancestral genomes many generations previously.

One issue that we have with Woese's view of early cellular evolution is that he does not clearly distinguish between two different phenomena that would occur if the HGT rate were very large: the loss of coherence of the species and the loss of coherence of the genome. Our model clearly shows that when *h *is large, there are no groups of genomes that form coherent species that are separable from other species. Reduction of *h *leads to the "origin of species" composed of coherent groups of genomes. In view of the title of Darwin's famous book, it seems appropriate to name this transition the Darwinian Threshold. However, this transition is not the origin of Darwinian evolution. As we just pointed out, even before the Darwinian Threshold, genomes contain groups of genes that remain coherent over many generations and can be selected as a team. Although horizontal transmission is very important prior to the Darwinian Threshold, this does not mean that vertical transmission is irrelevant. In fact, the majority of transmission is still vertical. In order to get to the point of loss of coherence of genomes where vertical inheritance becomes irrelevant, we would need to turn up *v *and *h *to values very much larger than any that we have considered in these simulations. It is not clear to us whether such a situation could ever have existed, as problems such as the spread of parasites [26] would indeed be extremely severe in this situation. On the other hand, the situation where the species lose coherence but genomes remain coherent makes sense in the framework of our model, and the high level of inconsistency of gene trees at the root of the tree of life that is seen in real sequence data suggests that this situation could well have existed in the early stages of prokaryotic evolution.

## Conclusions

Given that the model presented here predicts that organisms will evolve towards low rates of HGT, it is necessary to consider why there is still a significant rate of HGT observed in modern prokaryotes. In the case of transduction and conjugation, it may be that there is an arms race between cells and mobile genetic elements, and that cells are unable to prevent infection by viruses and plasmids that are evolving for their own selfish ends. Given that foreign DNA is entering the cell by these means, it may be that HGT occurs as an inevitable consequence. This arms race is not captured in the present model. In the case of transformation, imported DNA may be used as a food source [27] or imported sequences from another member of the population may be used as template for gene repair and homologous recombination [28]. These two potential advantages of transformation have nothing to do with HGT *per se*, but if higher DNA import rate were selected for these reasons, this would increase the rate of HGT as a side effect. If an explicit benefit were added to the model representing the nutrient value of imported DNA, it is likely that the HGT rate would have a non-zero optimal value, even if *v *were very low.

It should be remembered that when we observe examples of a gene with an apparently beneficial function that appears to have spread horizontally, this may be very rare in comparison to transfer of deleterious genes or junk DNA. The few beneficial cases will have important consequences for adaptation of the organism. Nevertheless, organisms cannot cherry-pick the best genes. If, on average, a transferred gene is deleterious, selection should lead to reduction of the HGT rate.

The rate of gene gain and loss among groups of closely related genomes is staggeringly high [29], but it is likely that most of the genes gained are not beneficial and are deleted again relatively rapidly [29,30]. A rapid rate of gain and loss of non-beneficial genes is consistent with the observation that the size of the pan-genome is very much larger than the size of any one genome [8,9]. If the pan-genome were very large and all new genes were advantageous, then selection would cause genomes to get larger and larger. In fact, bacterial genomes vary considerably in size, and larger genomes contain a higher fraction of genes that have been acquired from phylogenetically distant sources [31], but it seems unlikely that there has been a general increasing trend in genome size since the time of the emergence of the major lineages of prokaryotes. If the gene deletion rate, *v*, is not too large in modern organisms, the only way that the pan-genome can be large whilst genomes remain small is if most genes in the pan-genome would not be beneficial if inserted. With this in mind, it would be interesting to add a category of junk genes to our model that would contribute a cost, *c*, in the fitness, but no selective advantage. The rate of origination, *u*, of the junk genes might be much larger than that for beneficial genes, so that the total pan-genome would become very large, but the pan-genome of beneficial genes would remain comparable to the single genome size (as it is in Figure 2 for the simulation where all genes are beneficial and *u *is small).

There are few population genetics theories that include HGT; however, a theoretical calculation of the probability of spread of a single new gene through a population by HGT is possible [32]. To understand genome evolution, we will need theories that apply to gain and loss of multiple genes. Population genetics modelling of systems with variable genome size, poorly-defined species boundaries and HGT is still in its infancy, and is mathematically complex, even for the neutral case [33]. The model we have introduced here incorporates the simplest conceivable fitness function. Nevertheless, it is sufficient to shed light on key questions related to the evolution of HGT both in the early stages of life on Earth and in modern organisms.

## Competing interests

The authors declare that they have no competing interests.

## Authors' contributions

AAV ran the evolutionary simulations and wrote the paper. PGH designed the evolutionary model and wrote the paper.

## Reviewer 1

**Eugene V. Koonin - National Center of Biotechnology Information, Bethesda, Maryland**.

I believe that this is a very useful, potentially important paper that addresses a key problem in evolutionary biology, namely, the selective forces that affect the rate of HGT, using a straightforward and transparent mathematical modeling approach. The results are intuitively plausible, biologically relevant and I think overall correct: selection acts to increase the HGT rate if the accuracy of replication (here defined as the rate of gene deletion/loss) is low but then, higher accuracy evolves, and after crossing some threshold, selection acts to lower HGT. This is not all - Vogan and Higgs make another observation of no lesser importance: the model and its interpretation clearly distinguish, I believe for the first time, between the coherence of genomes and coherence of species. This distinction is logically unassailable and, once realized, should remove most of the confusion that is habitually associated with the concept of "rampant" HGT at the early stages of evolution.

I have one point of conceptual disagreement about the "Darwinian threshold". Vogan and Higgs write: "In view of the title of Darwin's famous book, it seems appropriate to name this transition the Darwinian Threshold. However, this transition is not the origin of Darwinian evolution." Indeed, in view of the title of Darwin's book, the phrase "Darwinian threshold" might seem appropriate. The problem, however, is that (short version of) Darwin's title is extremely misleading - indeed, given the unprecedented importance of the book, this might be the most misleading book title in history. In his book, Darwin does not explain the mechanism of speciation in any satisfactory manner. What he does explain, is the mechanism of evolution by means of natural selection, and this concept survived, even if it is only a part of the edifice of modern evolutionary biology. Therefore, I find the phrase Darwinian threshold to be extremely unfortunate as the name for the evolutionary transition associated with the emergence of species. Even if largely a semantic issue, this adds a lot to the confusion about the early stages of evolution. Vogan and Higgs correctly indicate that this threshold is not at all the beginning of Darwinian evolution (how could it be, indeed?!) but nevertheless stick with the unfortunate phrase.

*Author response: It is a fair comment that Darwin did not discuss speciation, but we still need a name for the transition, and this is the name that Woese gave it. The reviewer is in agreement with us about what actually occurs at the threshold, and about the importance of the distinction between the coherence of genomes and the coherence of species*.

The main comment on presentation is that, when speaking of replication accuracy, the authors should be very clear that here they deal only with the gene loss rate. Most people by default think of replication accuracy as the rate of single nucleotide mutations, so this needs to be clearly distinguished. More substantially, I wonder if it might be possible to somehow relate the model to empirically observed gene loss rates? Certainly, this would further increase the value of this work.

*Author response: Clearly we do not want to complicate the model to the level of single nucleotide mutations. One possible addition would be to include a rate of deleterious mutation that creates non-functional genes that do not contribute to fitness. We would expect deleterious mutations to play a similar role to gene deletions in this model. A paragraph was added in the methods section that discusses factors omitted from the model, and acknowledges that the model only includes gene loss and not single nucleotide mutations*.

The following sentence in the abstract "In contrast, if the accuracy of genome replication is high, as in modern prokaryotes, then HGT is unfavourable" and many similar statements in the text appear quite unfortunate. The impression made by these statements is that all HGT events are unfavourable, which would be ridiculous, and of course, the authors realize perfectly well that this is not the case, and say so. What is unfavourable, is a high rate of HGT not all and any HGT. This should be made crystal clear. In the same vein but more to the substance of the matter (here is the issue that seems important to me), I actually believe that selection acts to reduce but not eliminate HGT. More specifically, inasmuch as gene loss rate is certainly greater than zero, selection acts to reduce HGT to the extent that it more or less precisely balances the loss rate. Further decrease in HGT generally would unfavourable because a genome reduction ratchet would be set in action. Actually, in the case of intracellular parasites, when the HGT rate is dramatically reduced, we observe just such a ratchet. The most reduced of these parasites seem to be evolving towards the organelle status and then, in all likelihood, towards elimination. I wonder if the model developed in this paper has anything to say on that account, but even if not, it would be useful to discuss this problem.

*Author response: We changed the abstract to say that HGT is, on average, unfavourable in these circumstances. However, for the lowest gene loss rate in our simulations, we spent some time trying to ascertain whether there was some very small value of h that gave a higher mean fitness than h = 0. Within the limits of accuracy of our simulations, the optimum was indistinguishable from h = 0. Whether the optimum h is zero in the real world is a separate question, but it is perfectly possible that it is extremely small. HGT is not the only thing that balances gene loss. Gene duplication and neo- or sub-functionalization also occur in real genomes (factors that are omitted from our model). Also, even if the rate of occurrence of gene deletions in individual genomes is appreciable, the rate of fixation of a gene deletion in the population may be negligible in bacteria with large effective population sizes. Although it is true that the rate of insertion of genes via HGT is likely to be reduced in intracellular bacteria, it seems to us that this is not the main cause of the reduction of genome size that occurs in these organisms. One reason for loss of genes in intracellular bacteria may be that genes that were required for a free-living lifestyle are no longer needed in the environment of the host cell and are no longer maintained by selection. Secondly, bacteria that switch to an intracellular lifestyle experience a reduction in effective population size; hence there is a much higher chance of fixation of a gene deletion, even if the gene is still useful. Thirdly, if a newly functional advantageous gene arises within a genome, it is less likely to be fixed in a population with small effective population size*.

## Reviewer 2

Anthony Poole - Stockholm University

This article investigates the possible role of horizontal gene transfer in early evolution, and reports that, under accurate replication, lower rates of HGT are optimal. Taking a modelling approach to the genetic make-up of early systems seems to me to be the most productive way of addressing speculation on the role of HGT in early evolution. The analyses reported by Vogan and Higgs make a number of assumptions that are worth considering further. In the model, there is a selective advantage *s *for each gene type represented in a genome, and a lower cost *c *for every gene present. Because *s *>*c*, all non-duplicate genes are beneficial, but duplicate copies are rendered deleterious, with cost *c*. Genes are lost with probability *v*, an event that is far more frequent than the emergence of new gene types *u*, and *h *is the mean number of genes gained per genome by HGT. A consequence of this set up is that HGT will on the whole be advantageous because genes are lost *v*, and new genes are advantageous *s*. An obvious comment is that because gene types are generally advantageous, where deletion rates *v *are extremely low, HGT should be marginalised. This leads to the interesting conclusion that, using the gene-loss parameter *v *as a proxy for mutational loss of function, the higher fidelity a system is, the lower the optimal value of *h*. Under high *v*, HGT becomes essential because this counters loss of fitness arising from reduction in gene types.

The work presented here is interesting in that it attempts to address the key question of whether rates of gene transfer would have been higher at the earliest stages of evolution. The assumption that early genetic systems were subject to higher rates of transfer seems to have been widely adopted, but Vogan and Higgs rightfully recognise that this assumption does need to be scrutinised. The analysis presented by the authors does do this, and their result is therefore bound to be of broad interest. However, an important omission in this model is the association of genes with specific functions. Because all non-redundant genes are advantageous, and all combinations are allowed, in contrast to models like the hypercycle [19] or the Stochastic Corrector Model [21], the model presented here has no requirement for a specific combination of genes to be present for the system (i.e. cells) to operate. This amounts to an extreme form of functional redundancy (any unique gene will do), making the model rather abstract.

Another area where the analysis presented does need to be taken further concerns how redundancy is treated. In the model presented here, redundancy is disadvantageous. A significant body of literature argues that redundancy is likely for low-fidelity systems, since it buffers against gene loss. Previous authors have shown this using modelling-based approaches [22,23]. The model presented here essentially replaces redundancy with gene receipt (because any unique gene will do as a counter to gene loss). Making redundant copies deleterious therefore ignores previous results arguing that redundancy will be important in systems with low fidelity, both as it can buffer against mutation and because selection can operate between variants even at high copy number. It also seems clear that this model does create the conditions wherein an alternative process favours a different form of redundancy. It would therefore be interesting to know what happens if one requires some combination of gene types to be present, and, rather than a model wherein 'any non-redundant gene type will do', the authors instead required specific gene types to replace lost genes.

A third issue is that further examination of the possible role of HGT in early evolution may require a model that incorporates different levels of selection. While the authors discuss this in their manuscript, their model focuses only on selection at the level of the cell. Expanding discussion to models where two levels of selection can operate, such as the Stochastic Corrector Model [21,24] would therefore be valuable.

*Author response: We have added an additional paragraph at the end of the methods section that refers to hypercycles and the stochastic corrector model (SCM). The SCM deals with a situation in which each gene is treated as a separate molecule segregating randomly in the cell. In this situation, the possibility of loss of a gene from one of the daughters is large. If the cell has multiple copies of a gene prior to division, there is a larger chance that both daughter cells will inherit at least one copy of the gene. The SCM is important because it shows the population can remain viable as a whole for some parameter values, even if some inviable cells are created. It also shows that a population can resist invasion by parasites if there is sufficient selection between cells to overcome the competition between molecules inside the cell. Furthermore, variants of the SCM have shown that linked genes can be selected in this model *[25], *and this is an important step to get from a situation of a protocell with a bag of independent and potentially competing genes to a modern cell with a team of linked, cooperating genes that replicate together on large chromosomes. Thus, in our view, the SCM is important because it shows that even the simplest bag of genes can overcome key problems. However, it also highlights how inefficient a cell with independent randomly-segregating genes would be. It is difficult to imagine a protocell of this form with more than a handful of different kinds of gene. There would be huge selection pressure to create linked chromosomes and to develop mechanisms that achieve better than random segregation. In this paper, we want to treat organisms that have reached the level of complexity of cells at the time of the LUCA, and we suppose that these cells would have had at least a few hundred genes, or maybe a few thousand as with modern bacteria. We suppose that these cells had already improved well beyond the bag of genes stage. Our model supposes that the genome already consists of linked chromosomes and that gene loss is occurring principally due to deletions during replication rather than by segregational loss*.

*The model of Koch *[23], *which was raised by the reviewer, assumes there are n copies of a gene, that this is duplicated to 2n, and that exactly n of these are passed on to each daughter cell. He then considers the possibility of fixation of a new allele for this gene. However, by assuming an exact division with n copies each side, this model disallows the possibility of loss of the gene. So this model seems to miss the point about random segregation*.

*The reviewer also comments on the possible advantages of redundant genes. One advantage is that the probability that more than one copy will be lost (by deletion, mutation or inaccurate segregation) in one generation is less that the probability that a single copy will be lost. Thus if a parent cell contains duplicates it will have a higher proportion of viable daughter cells. This buffering effect gives an indirect benefit to duplicates that can affect offspring fitness but not parental cell fitness. However, larger cells with many duplicate copies will take longer to replicate. So redundant genes create a direct cost to the fitness of the parent cell. Our model includes both of these features. If a parent cell has n copies of a gene, the probability that all these copies are deleted is v^n^, so there is a high probability that at least one copy is retained if n > 1 initially. Nevertheless there is a cost c for each duplicate. It can be seen in our results that, for the larger values of v, successful genomes possess duplicate genes. For example, in *Figure 2$\bar{n}$*is much greater than *$\bar{n_{types}}$*when h is close to its optimum (around 0.6). Thus, our model already includes the buffering effect that the reviewer comments on*.

*There is another possibility that a duplicate gene could give a direct fitness benefit to the parent cell because it creates an increased dosage of the gene product. Many modern bacterial genomes possess duplicate genes for rRNA and tRNAs, presumably because this allows for more rapid transcription of the large number of rRNA and tRNA molecules that are required in the cell. However, there are very few duplicate copies of protein-coding genes in bacteria, even when large numbers of protein copies are required (as with ribosomal proteins). This is presumably because a large number of proteins can be translated from a small number of mRNAs, and a single gene copy is sufficient to transcribe the required number of mRNAs. Direct benefits of duplicates appear to be rare in prokaryotes, and our model does not include any direct benefits*.

The concern I raised [26] about the 'communal ancestor' model was that it isn't clear on levels of selection, and, in the form presented by Woese, it seems to be predicated on naive group selection arguments (genes cooperating by default and in the absence of parasites - a clear parallel exists with the shortcomings of the hypercycle [34]. In this respect, the model presented by Vogan and Higgs does not address the problem of "communal" gene sets because all non-redundant gene combinations are equivalent, and no genes are ever detrimental. So if I understand correctly, the only reason for transfer being favoured at all in this model seems to be because v is not zero and all genes are functionally redundant. Cooperating gene sets must have emerged at some point, and explaining their emergence from a system that is vulnerable to gene-level selection (i.e. parasites) is a crucial part of the evolutionary transition to cells [35]. I note however that the authors state in their discussion that, under a model with parasites and gene disruptions (via genome insertions), HGT would be less advantageous than in the case considered.

*Author response: The reviewer's use of the word redundancy is misleading. Each new gene in our model represents a gene with a new function that is not redundant with previous genes. For simplicity, we suppose each new gene gives the same fitness benefit, but this is not the same as redundancy. The reason that HGT is favoured in the model has nothing to do with redundancy. The advantages of HGT are that it allows a cell to acquire beneficial genes that were originally evolved in another cell and it allows cells to regain genes that were deleted. We agree that our model does not deal with the levels of selection issue. We have argued above that this problem must already have been solved by creating linked chromosomes prior to the stage considered in our model. It is obvious that if we added horizontally transmitted parasites and gene disruptions due to HGT, then HGT would be less advantageous than in the present model. It is already the case that there is selection to reduce the rate of HGT in the present model. Therefore these additional effects would just provide additional selection to reduce HGT, and would not qualitatively change the results*.

## Reviewer 3

**J. Peter Gogarten, Department of Molecular and Cell Biology, University of Connecticut, USA**.

The manuscript by Aaron Vogan and Paul Higgs describes a simulation of genome evolution with the aim to characterize the processes that occurred at the emergence of coherent genomes. The simulations illuminate a valid point concerning the Darwinian threshold: accurate replication removes the necessity for high levels of gene transfer. While I agree with this general conclusion, I have a few questions, comments and suggestions. Some of these might seem unfair to invoke in face of the straightforward and limited simulations; however, the authors themselves take a broad view in discussing their findings.

The performed simulations describe a sympatric process, corresponding to sympatric speciation, if viewed as speciation. I do not know how frequent this process is in extant microorganisms, but allopatric speciation, driven by geographical division of a population, appears to be frequent among extant organisms. Its requirement is that gene flow across the geographical barrier is low compared to within group transfer. Geographical isolation could split a population of communal entities (precells, progenotes, or hypercycles) before crossing the Darwinian threshold. The splitting of a population into separate lineages through an allopatric process depends on barriers to gene flow between the separate lineages, and not on the question whether distinct genome genealogies exist within a single lineage.

*Author response: In a more complex model, we might have considered geographical barriers. However, the simple model we studied already shows the Darwinian threshold effect that we are interested in, so additional complexities are not required at this point*.

As illustrated by the above comment, some of my uneasiness in reading the article stems from the fact the authors do not always distinguish between within species/population gene transfer, that often results in homologous recombination, and gene transfer between divergent organisms often resulting in non homologous recombination. I readily admit that the distinction between these processes is not always clear - in some instances gene transfer between species can result in homologous recombination and the transferred gene, although very divergent in sequence, can be functionally equivalent to alleles in a population [36]. The simulations by Vogan and Higgs treat gene transfer as equivalent to non-homologous recombination (the transferred gene is added to the recipient genome); however, in the discussion they also include processes that result in homologous recombination, the latter process is equivalent to sex in eukaryotes in that it allows organisms to combine beneficial mutations that occurred in different genealogies [37], and prevents Muller's ratchet [38] from operating. The occurrence of homologous recombination in bacterial and archaeal species has been amply documented (see [39] for review). While the benefits and costs of sex continue to be debated, the widespread occurrence of sex suggests that the benefits are substantial. If within population transfers resulting in homologous recombination are included under HGT, then the generalized statement "if the accuracy of genome replication is high, then HGT is unfavourable" does not appear warranted. It might be true for the performed simulations, but not as a general statement for biology.

*Author response: We hope it is clear that we are modelling gene gain and loss, which is non-homologous, and we are not modelling homologous recombination because we are not considering variations between alleles for the same gene. The points above are relevant at the level of evolution of a single gene. Our model is working at the whole-genome level. The statement in the abstract is now changed to "if the gene loss rate is lower, then HGT is, on average, unfavourable"*.

Evolution constitutes a strange loop where the process by which evolution occurs can be subject to selection pressure. Thus parameters such as mutation and gene transfer rates might be subject to selection and change over evolutionary time. It is tempting to speculate that the observation that gene transfer is biased towards transfer between closely related organisms [36,40,41] might reflect the fact that transfer between more divergent organisms is more frequently detrimental to the recipient, possibly because more molecular parasites might be acquired through these transfers. However, this bias might also reflect the simple fact that more mechanisms exist for transfer between closely related organisms, and that the transferred gene has a better chance of being integrated into the recipients regulatory and metabolic networks if it originated from an organisms with similar sequence biases and regulatory signals. The question "if HGT is on average beneficial to the organism" needs to be considered for the different types of gene transfer (see the previous paragraph). Another way the question needs to be differentiated is the meaning of "beneficial to the organism". Sometime ago Jeff Townsend and I proposed the neutral or nearly neutral theory of HGT [42] in analogy to Kimura's neutral theory. According to our proposal, most transferred genes found in a population are neutral or nearly neutral to the recipient. This does not mean that all transfers are neutral, many, or even most transfers may be detrimental to the recipient, but because they lower the fitness of the recipient, these genes are quickly eliminated from the population. Neutral, or nearly neutral transferred genes may be fixed in the population through drift, but as Lawrence and Ochman found, these genes often do not persist in the recipient lineage over long periods of time [43]. In addition, most of the genes identified as acquired through HGT in a genome have not been fixed in the population, and most of them never will be. The hypothesis that most of the transferred genes observed in a population are neutral or nearly neutral does not say that most transferred genes are neutral, most of the transferred genes might be detrimental, but because they are subject to purifying selection they are not frequently encountered in the population.

*Author response: The question of whether transfer occurs more frequently between closely related species is very interesting, and it is easy to envisage more complex models where the either the attempt rate or the successful incorporation rate of HGT is larger for more similar genomes. Also, we agree that many newly gained genes are not beneficial and will not spread widely. This point is already discussed in our conclusion, and we already pointed out that it would be useful to extend our model by including a category of non-functional genes. Addition of non-functional genes would allow the pan-genome to become much larger, without significantly increasing the size of individual genomes. However, addition of non-functional genes would also make HGT less favourable than the present case. Therefore, once again, this would not change the central result of our model that there is selection to reduce the rate of HGT*.

The benefit or detriment of gene transfer to a population is a different matter. The authors claim that their simulations provide evidence against a statement in [1] that barriers to HGT from divergent organisms might not be of significant selective advantage to the group. Or in other words, according to [1] preventing rare transfers, even if most were detrimental to the recipient, would not create a significant selective advantage for the group. An example might illustrate my reasoning (I don't claim to speak for my co-authors). Assume a population of 10^9 ^organisms; 200 of these acquire a gene from a divergent organisms. In half of the cases, the recipient is less fit, and these genes will be rapidly eliminated from the population, in 99 cases the transferred genes might be nearly neutral and most of them will be eliminated from the population due to drift. Even if a nearly neutral gene drifts to fixation in the population, as long as it is not a molecular parasite with mechanisms for its own long term survival, the gene will be subject to the deletion bias prevailing in bacterial genomes [44], and the nearly neutral gene will be eliminated on the long run. The rare advantageous gene might open up new resources, be rapidly fixed in the population, and/or spawn divergence into a new ecological niche. Even though in the example half of the acquired genes are detrimental, the fitness of the population is hardly affected negatively. The difference between this reasoning [1] and the simulations by Vogan and Higgs could be that in the simulation gene transfer between organisms is not rare, rather in the simulations transferred genes accumulate in the genomes of the population decreasing the average fitness of its members. In real life the ballooning of genome content is prevented by the deletion bias, even though the adaptive value of the deletion bias might be the inactivation of molecular parasites and not in streamlining the genome.

Thus I still think that if gene acquisition from divergent species is rare, then effective selection for barriers to transfer would not occur. To the contrary, the rare transfers that provide a selective advantage, and that are driven to fixation in the existing population, or that lead to the founding of a new population in a different niche conceivably could select for mechanism that enable HGT events between divergent organisms. Some of these HGTs have changed the face of this planet, e.g., the two photosystems working in series in oxygen producing photosynthesis [45,46], or the pathway that allows methane production from acetate in Methanosarcina [47]. It is tempting to speculate that the creation of new metabolic pathways and capabilities was a driving force to select for and maintain HGT mechanism. While the idea of gene sharing mechanism providing an adaptive advantage is philosophically appealing to me, I do not know of any evidence that the transfer mechanisms between divergent organisms are under purifying selection due to the selective advantage provided to the recipient (this is different for within group transfer, where the existence of Gene Transfer Agents whose sequences appear to be under purifying selection suggests selection for the gene transfer machinery [48]). Furthermore, the transfer of genes beneficial to the recipient (antibiotic or heavy metal resistance genes) often can be explained from the selfish gene point of view rather than assuming group selection in favour of HGT [49]. It appears possible that these transfer events, however big their impact on the evolution of our biosphere, occurred as by products of other processes, such as uptake of DNA as a nutrient [27] as detailed by Vogan and Higgs, or as a by-product of the propagation of molecular parasites and viruses.

*Author response: The argument above seems to rest on whether HGT is 'rare' or 'common'. If it is as rare as in the example above, there will be almost no selection either way on the HGT rate because most individuals will not experience HGT. But Woese's proposal was that, in the early stages, HGT was common. We have shown that this makes sense if vertical inheritance is inaccurate, because high HGT rates are then favoured. But our model shows that when vertical inheritance becomes more accurate, there will be selection to reduce HGT. This would lead to the state described in this example, where HGT is too rare to be selected against. Thus, if we accept Woese's view that HGT was initially very common, there must already have been selection against it to reduce it to the levels that we see now*.

*We agree that rare advantageous cases of HGT can be important in evolutionary history, as in the examples that the reviewer gives. We already stated in our conclusions that "the few beneficial cases will have important consequences for adaptation of the organism". We also already said that the remaining low level of HGT could be due to selfish genes (molecular parasites and viruses) or selection for uptake of DNA as a nutrient. So the reviewer seems to be largely in agreement with us*.

Vogan and Higgs see the high level of inconsistency at the root of the tree of life as an indication for high levels of gene transfer at the time of the Last Universal Common Ancestor (LUCA). I do not agree. At the time of LUCA the use of the 20 canonical amino acids found in modern proteins, the ribosome, signal recognition particles, and chemisosmotic coupling had already been established [50]. While not all of the molecules present in all extant organisms were present in the same organismal common ancestor [51], the phylogenies of the listed traits are congruent and rooting them using ancient paralogs (see [52] for a review) or the echo from the assembly of the genetic code [53] consistently places the root between the archaea and bacteria, with the eukaryotic nucleocytoplasm being the sister to the archaea (which in the different analyses are recovered either as mono- or paraphyletic). In contrast to the statement in the manuscript by Vogan and Higgs, the deepest split in the tree of life is recovered with a surprising consistency, even analyses based on genome content [54] and midpoint rooting of the rather frequently transferred aminoacyl tRNA synthetases [55] place the root of the genome network in the same place. Together these observations (the inferred characteristics at the root of the tree and the congruence of phylogenetic patterns) suggest that LUCA had already crossed the Darwinian threshold.

*Author response: Our paper presents a theoretical model only and therefore we cannot add to the debate on the precise nature of the LUCA. For our purposes, it does not matter whether the Darwinian threshold occurred at the time of the LUCA or prior to this*.
